# Exploring the Blood Biomarkers and Potential Therapeutic Agents for Human Acute Mountain Sickness Based on Transcriptomic Analysis, Inflammatory Infiltrates and Molecular Docking

**DOI:** 10.3390/ijms252011311

**Published:** 2024-10-21

**Authors:** Jiayi Yan, Zhuo Zhang, Yunxuan Ge, Junru Chen, Yue Gao, Boli Zhang

**Affiliations:** 1 Institute of Traditional Chinese Medicine, Tianjin University of Traditional Chinese Medicine, Tianjin 301617, China; yjy19951025@163.com; 2 Department of Pharmaceutical Sciences, Beijing Institute of Radiation Medicine, Beijing 100080, China; zhangzhuo000@yeah.net (Z.Z.); luckwin@yeah.net (Y.G.); 15088094928@163.com (J.C.); 3 School of Pharmacy, Guangdong Pharmaceutical University, Guangzhou 510006, China

**Keywords:** AMS, bioinformatics, inflammatory infiltrates, molecular docking

## Abstract

A high-altitude, low-pressure hypoxic environment has severe effects on the health and work efficiency of its residents, and inadequate preventive measures and adaptive training may lead to the occurrence of AMS. Acute exposure to hypoxia conditions can have a less-favorable physiological effect on the human immune system. However, the regulation of the immune system in high-altitude environments is extremely complex and remains elusive. This study integrated system bioinformatics methods to screen for changes in immune cell subtypes and their associated targets. It also sought potential therapeutically effective natural compound candidates. The present study observed that monocytes, M1 macrophages and NK cells play a crucial role in the inflammatory response in AMS. IL15RA, CD5, TNFSF13B, IL21R, JAK2 and CXCR3 were identified as hub genes, and JAK2 was positively correlated with monocytes; TNFSF13B was positively correlated with NK cells. The natural compound monomers of jasminoidin and isoliquiritigenin exhibited good binding affinity with JAK2, while dicumarol and artemotil exhibited good binding affinity with TNFSF13B, and all are expected to become a potential therapeutic agents.

## 1. Introduction

Acute hypoxia will lead to a cascade of physiological changes that affect multiple systems in the body. In severe cases, it can result in high-altitude pulmonary edema (HAPE), high-altitude cerebral edema (HACE) and high-altitude pulmonary hypertension [1,2]. Acute exposure to hypoxia conditions can have a less-favorable physiological effect on the human immune system. The immune system’s regulation is a dynamic and multifaceted process involving numerous cell types, signaling molecules and feedback mechanisms. Decreased oxygen concentration (hypoxia) is a critical factor that influences the immune system in various ways. Recent studies have shown that CRP, IL-1β and IL-6 are increased in individuals acutely exposed to hypoxia [3]. The combination of increased pro-inflammatory cytokines and increased blood vessel permeability leads to vasogenic edema. A human study showed that during acute high-altitude exposure, the expressions of many inflammatory factors in peripheral blood were upregulated, among which the genes FASLG and SMAD7 were associated with AMS scores and SpO2 levels [4]. There was a lot of infiltration of neutrophils and macrophages in the pulmonary, myocardium and hippocampal tissue in rat and mouse hypoxia models [2,5,6]. In a high-altitude-induced ulcerative colitis model, the levels of inflammatory factors IL-17, TNF-α and IFN-γ in the colon, spleen and mesenteric lymph nodes and the numbers of Th1 and Th17 cells in the spleens were significantly increased [7]. The molecular signaling pathways that regulate inflammation and the response to hypoxia share significant crosstalk [4]. Under hypoxic conditions, the activity of PHD enzymes is inhibited and the IKK complex can proceed to remove IkB from NF-κB, which in turn increases its rate of translocation to the nucleus and upregulates a series of inflammatory gene expressions [8]. Due to various factors, including different rates of ascent and durations of hypoxia, results may exhibit contradictions or inconsistencies, and there is scant literature on the inflammatory markers and the trends of changes in different subtypes of immune cells in acute mountain sickness.

In the present study, we downloaded microarray datasets from the GEO database and screened high-altitude hypoxia-associated genes. The identified DEGs were analyzed by Gene Ontology (GO) and Kyoto Encyclopedia of Genes and Genomes (KEGG) pathway analysis. Cytoscape (3.10.2) visualized the PPI network and identified clusters and hub genes. In addition, the CIBERSORT algorithm was used to analyze the abundance of 22 kinds of immune cells and identify potential immune cell-related biomarkers along with their corresponding natural compound monomers, further testing them using the molecular docking method and aiming to provide insights for clinical medication use.

## 2. Results

### 2.1. Identification of Common DEGs

**Figure 1:**
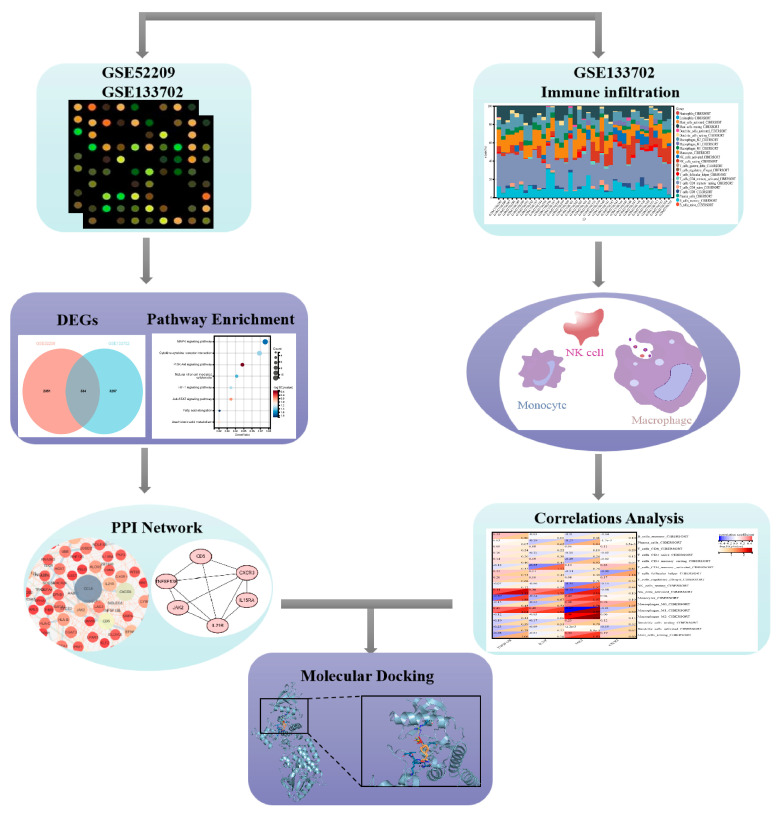
Flow chart.

**Figure 2:**
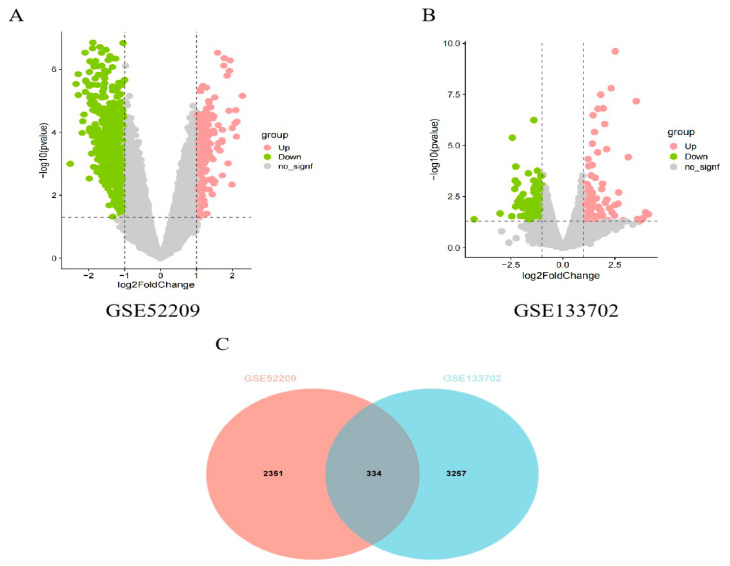
Identification of common DEGs. (**A**,**B**) Volcano plot revealing DEGs between the control group and HH-Day. (**C**) A total of 334 common DEGs were identified after taking the intersection of the DEGs in GSE52209 and GSE133702.

**Figure 3:**
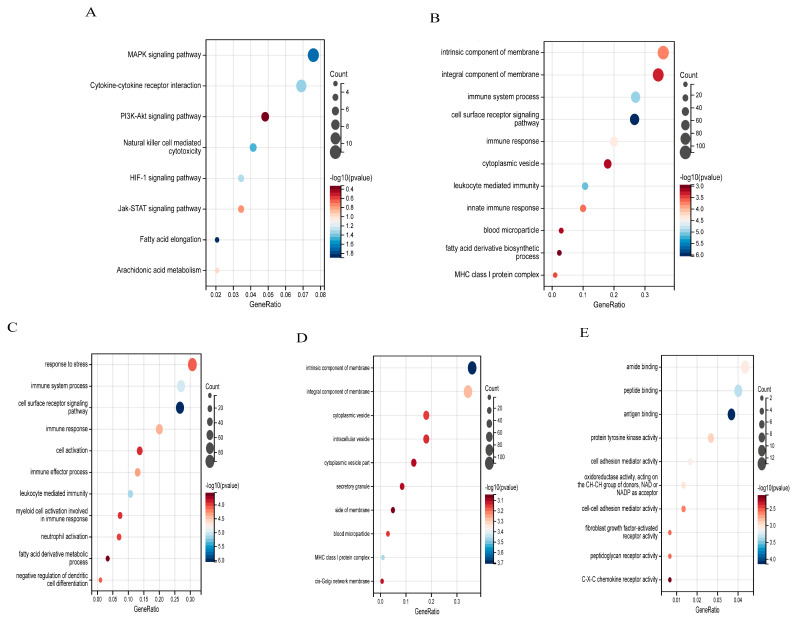
Functional enrichment analysis of the common DEGs. (**A**) KEGG enrichment analysis of the top eight enriched pathways. (**B**–**E**) Gene Ontology (GO) analysis.

The bioinformatics analysis of this study was carried out according to Figure 1. As shown in Figure 2A, there are 2685 DEGs in the high-altitude pulmonary edema patients compared with the healthy controls in GSE52209 dataset; 1710 of them are upregulated and 975 are downregulated. There are 3591 DEGs that were screened between the control group and the HH group in the GSE133702 dataset; 1948 of them are upregulated and 1643 are downregulated (Figure 2B). The Venn diagram illustrates the common genes between the two datasets. A total of 334 common genes were identified. To obtain a deeper insight into the functions of these common genes, we performed functional enrichment analyses using the GO and KEGG databases. The results revealed that the common genes were closely associated with processes such as humoral immune response, ATP synthesis and gas transport. KEGG pathway analysis showed that AMS exhibited increased activity in the MAPK, PI3K-Akt and JAK-STAT signaling pathways (Figure 3).

### 2.2. PPI Network Construction and Hub Gene Identification

**Figure 4:**
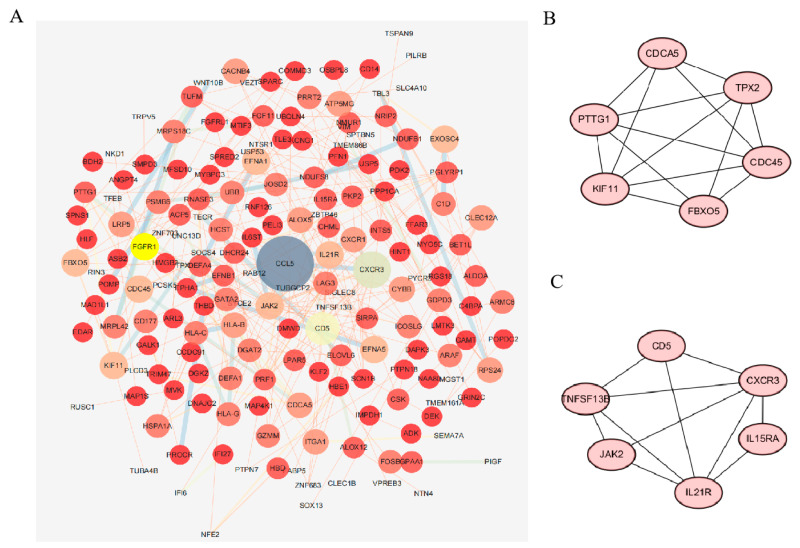
PPI network construction. (**A**) PPI network of common DEGs. The size of the circle represents the degree. (**B**,**C**) Two key clusters were identified in the network based on MCODE.

The PPI network was first constructed in the STRING database to determine the interactions among the common DEGs. Cytoscape was used to visualize the common genes (Figure 4A), and the MCODE algorithm was used to find the closely connected protein groups in the target network, with the top two modules displayed in Figure 4B,C. Cluster 1 contained six potential hub genes, KIF11, CDCA5, CDC45, TPX2, FBXO5 and PTTG1, which belong to essential genes for life. Cluster 2 contained six potential hub genes, IL15RA, CD5, TNFSF13B, IL21R, JAK2 and CXCR3, which are highly enriched in inflammatory response pathways.

### 2.3. Immune Cell Infiltration of High-Altitude Hypoxia

**Figure 5:**
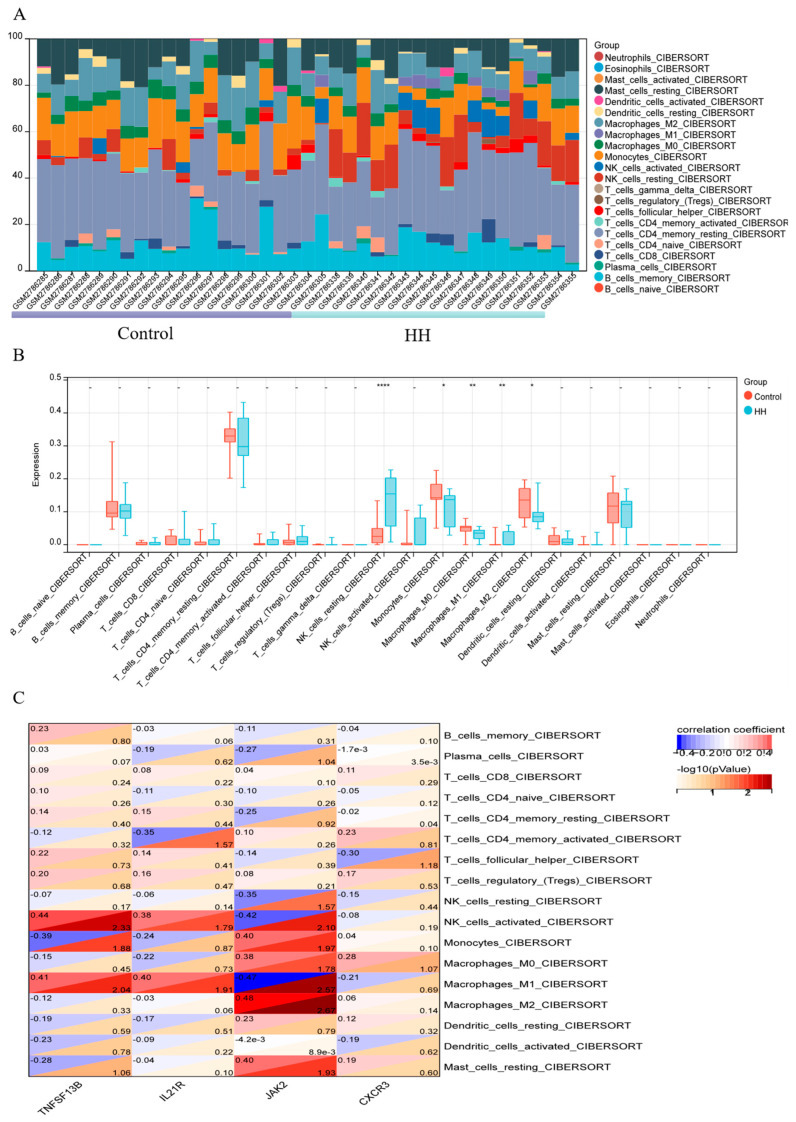
Profile of immune cells in different times of hypoxia exposure. (**A**) Immune cell content stacking plot; different colors indicate different immune cells. (**B**) This box plot shows the differences in immune infiltration between the two groups. (**C**) Correlation analysis of 22 types of immune cells. Red: positive correlation; blue: negative correlation.

As shown in the histograms (Figure 5A), the proportions of different subtypes of immune cells change following hypoxia on the plateau. Significant differences were demonstrated between the HH and control groups of five immune cell types (*p* < 0.05). After exposure to a hypobaric hypoxic environment for seven days, there were significant decreases in monocytes and M0 and M2 macrophages in the human peripheral blood, as well as significant increases in M1 macrophages and NK cells.

### 2.4. Correlations between Hub DEGs and Immune Cells

To further understand the role of these genes in immune infiltration, whether these hub genes were related to immune cell infiltration was evaluated by Pearson correlation. In Figure 5C, a positive correlation analysis shows that JAK2 is associated with the expression of monocytes (r = 0.4) and M0 macrophages (r = 0.48) and that TNFSF13B and activated NK cells are positively correlated (r = 0.44).

### 2.5. Validation of the Interaction of Natural Ingredients with Hub Genes

**Figure 6:**
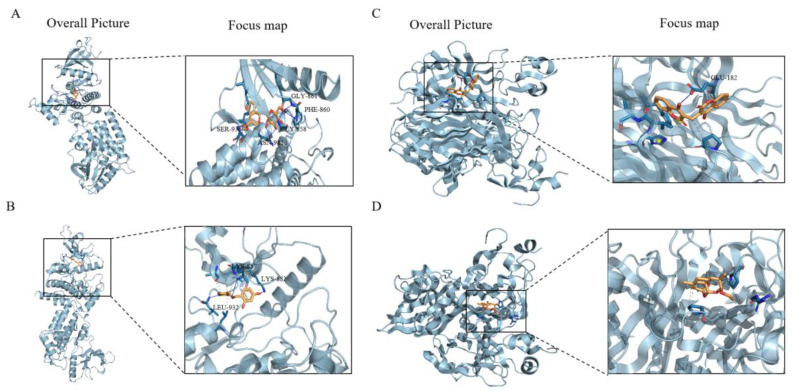
Molecular docking results of the main active compounds and the key targets: (**A**) JAK2 and jasminoidin; (**B**) JAK2 and isoliquiritigenin; (**C**) TNFSF13B and dicumarol; (**D**) TNFSF13B and artemotil. The yellow structure represents small molecules; Blue structure represents amino acid residues of targets.

In the present study, inflammation-related cluster target proteins were selected for molecular docking analysis and candidate natural compounds were selected from drug bank and HERB databases. According to the minimum binding energy, as shown in Table 1, jasminoidin forms hydrogen bonds with JAK2 at the GLY-858, PHE-860, GLY-861, SER-936 and ASN-981 positions; isoliquiritigenin forms hydrogen bonds with JAK2 at the LYS-857, LYS-882 and LEU-932 positions; and dicumarol forms hydrogen bonds with TNFSF13B at the GLU-182 position. The lengths of the hydrogen bonds between the compounds and targets are shown in Table 2. In Figure 6, we have used the visualization method to visualize the specific binding of different compounds to protein residues.

## 3. Discussion

A high-altitude, low-pressure hypoxic environment has severe effects on the health and work efficiency of its residents, and inadequate preventive measures and adaptive training may lead to the occurrence of AMS (acute mountain sickness). With different ascent methods and altitudes, the incidence of AMS has ranged from 10% to 93% [9]. Therefore, research on acute high-altitude hypoxia is urgent. The effects of low pressure and hypoxia on the human body manifest as multi-system and multi-organ involvement. Among these, the immune system is one of the most significantly affected. After oxygen deprivation, changes will occur in the number, homing, functions and metabolic reprogramming of immune cells. This study primarily investigated the changes in the number of immune cells in human peripheral blood following acute high-altitude hypoxia, identified the pathways involved and screened for corresponding biomarkers and potential natural product candidates with therapeutic effects.

The GSE52209 and GSE133702 datasets were included in this study, with the GSE52209 data derived from patients with high-altitude pulmonary edema and GSE133702 data selected from individuals three days after hypoxia exposure. In the study corresponding to these two datasets, key indicators such as blood pressure, heart rate and oxygen saturation in individuals three days after hypoxia were found to be significantly altered compared with a control group [10]. Therefore, these two datasets have significant clinical reference value for the screening of biomarkers for acute high-altitude hypoxia. The two datasets share 334 common differentially expressed genes. The PPI network illustrates the network relationships among the common genes. The MAPK signaling pathway, natural killer cell-mediated cytotoxicity, the JAK-STAT signaling pathway and the arachidonic acid metabolism pathway were closely associated with hypoxia stress. p38 MAPK and JNKs are activated by pro-inflammatory cytokines (PAMPs/DAMPs) [11]. Xing Fu found that lung tissue inflammation was associated with PI3K/AKT and MAPK signaling in an acute hypobaric hypoxia rat model [12]. A natural flavonoid was stably combined with p38, ERK1/2, JNK and NF-κB, showing cardioprotective effects against MI/RI in vivo [13]. The Janus kinase-signal transducer and transcription activator pathway (JAK-STAT) is critical for cellular signaling and regulates physiological and pathological processes such as inflammation and stress. SD rats exposed to acute hypobaric hypoxia for 1 h at a simulated altitude of 9144 m exhibited changes in the lung tissue involving the MAPK, p53 and JAK-STAT signaling pathways [14]. Arachidonic acid (AA) is an important structural lipid, found in phospholipids in the blood, the liver, the muscle and other organ systems, that represents a wide range of immune–physiological effects in animals [15]. AA is catalyzed by lipoxygenase (LPO) to produce the inflammatory mediators that are hydroxyeicosatetraenoic acids (HETEs). It was reported that arachidonic acid metabolism potentially promotes the spleen hypoxia response [16]. Interestingly, our experiments also found that after three days of hypoxia, the levels of arachidonic acid metabolite 12-HETE and 15-HETE in rat myocardia significantly increased. After the knocking down of the ALOX15 gene, the inflammatory infiltration in the rat myocardia decreased and the number of monocytes in the peripheral blood dropped (unpublished data). The MCODE plugin was used to cluster the common DEGs, with Cluster 1 consisting of essential human genes and Cluster 2 being related to inflammation. The genes enriched to the first Cluster (Cluster 1) are a group of essential genes that maintain key cellular functions, which are the most important components of an organism [17]. They are essential for the survival of the organism and for the maintenance of basic functions of the cell or tissue [18,19]. Motor protein KIF11, a kinesin, has functions that include chromosome localization, participation in various structural dynamics, protein binding, ATP binding, etc. [20,21,22,23]. CDCA5 and CDC45 are both cell-division cycle-associated proteins that are related to chromatin-binding activity [24] and essential for initiating DNA replication [25,26,27] and also play an important role in DNA repair [28]. TPX2 activates protein kinase activity and plays an important role in gene expression. It is also a microtubule nucleation factor that has a direct effect on the formation of the microtubule cytoskeleton and the assembly of the spindle in mitosis [29,30], and is a novel biomarker for the development of human cancers [31]. The role of FBXO5 involves protein kinase binding, cell cycle and mitosis [32,33], and it is also participating in DNA replication and DNA damage response [34,35,36], affecting chromosome stability [37]. PTTG1 protein plays a central role in chromosome stability, DNA damage and repair [38,39,40]. Therefore, these genes are generally central hubs for biological functions and are altered in expression during stress responses, disease development and other processes in the human body. Alterations in human essential genes under conditions of acute hypoxia are to be expected; however, our study reveals exactly which essential genes are significantly altered under such conditions. This is an important reference for the discovery and interpretation of hub genes, selection of biomarkers, and screening of therapeutic targets under acute hypoxic conditions. The Cluster 2 related to immunity, will be subjected to a correlation analysis with the results of immune infiltration. Moreover, we analyzed the functional pathways for both of the clusters’ genes (Figures S1 and S2). The pathways enriched for genes in the Cluster 1 are involved in Cell Cycle, Mitotic Metaphase and Anaphase, G1/S-Specific Transcription, M Phase, S Phase, and G1/S Transition. The results are consistent with the discussion of gene function above, and these pathways are closely related to the cell cycle, cell mitosis, transcription-translation, and protein binding. The pathways enriched for genes in the Cluster 2 are involved in Cytokine-cytokine receptor interaction, JAK-STAT signaling pathway, Intestinal immune network for IgA production, and Th17 cell differentiation. These pathways are intimately involved in regulation immune response, inflammation, cell growth, differentiation, and usually in response to an activating stimulus. These inflammation-associated molecules and pathways are significantly altered in response to stress or disease in the organism and are critical for immunity and host protection.

In this study, GSE103927 dataset was applied to further explore the landscape of immune infiltration in acute high altitude hypoxia. During acute hypoxia, changes in the number of immune cells are mainly concentrated in the innate immune cells. After seven days of hypoxia, the number of monocytes in the peripheral blood decreased, while the M1 type macrophages increased, which is related to the differentiation of monocytes into tissue macrophages following hypoxia. It was reported that the activation of HIF-1α can promote the differentiation of monocytes into tissue macrophages a process that plays a significant role in inflammation and tissue repair. The PHD2D4E;C127S suppressing pro-inflammatory functions of monocytes in response to hypoxia by down-modulation of signaling molecules p65, ATF2, STAT1, cJun and CXCR4 are a key factor in the hypoxic inflammatory response during this process [41]. During a stage of acute hypoxia for 7 days, the ratio of M1 to M2 macrophages increases, with M1 macrophages dominating and releasing pro-inflammatory cytokines that lead to focal and strip-like inflammatory infiltration in the tissue [12,42]. The JAK2 gene, which is positively correlated with M2 macrophages, is involved in the polarization process of macrophages and plays a crucial role in maintaining the homeostasis within macrophages [43,44]. Natural killer (NK) cells are immune cells capable of directly killing target cells without prior sensitization. Research indicates that hypoxic conditions can stimulate the proliferation of NK cells, potentially as a compensatory mechanism to increase their numbers in response to the stress of low oxygen levels. This proliferation is essential for maintaining effective immune surveillance. However, those researchers found that the cytolytic abilities of healthy donor (HD) NK cells were markedly impaired under hypoxic conditions, which was associated with a reduction in the secretion of lytic agents and receptor/ligand interactions [45,46]. However, another research report indicates that both acute and chronic exposure to an altitude of 5000m lead to an increase in the number of NK cells in peripheral blood, but the cytotoxic activity of NK cells is not affected by HA exposure [47]. In our study, the number of NK cells significantly increased after hypoxia, accompanied by the downregulation of the TNFSF13B. It has been reported that increases in TNFSF13B transcription lead to reductions in ILC1/NK cell differentiation [48], which is consistent with our findings.

In addition, we have identified certain natural compound monomers from public databases that exhibit good binding affinity with key genes. Jasminoidin (JA) is a type of iridoid glycoside compound, primarily derived from the Gardenia jasminoides Ellis plant, that has been shown to have protective effects in cases of cerebral ischemia–reperfusion and hepatic inflammation [49,50]. Isoliquiritigenin, a bioactive ingredient in Glycyrrhizae Radix, significantly downregulates the secretion of IL-1β, IL-6, TNF-α and MCP-1 in vitro and in vivo and inhibits the inflammation and fibrosis of macrophages [51]. There are also literature reports on the anti-inflammatory therapeutic effects of dicumarol and artemotil [52,53].

Regulation of the immune system in high-altitude environments is extremely complex. In addition to understanding the changes in the number of various types of immune cells, further investigation is needed to determine whether their functions are impaired after hypoxia as well as changes in cellular metabolism and energy production.

## 4. Materials and Methods

### 4.1. Data Collection and Preprocessing

We systematically searched high-altitude-related study Gene Expression Omnibus (GEO) datasets (http://www.ncbi.nlm.nih.gov/geo/, accessed on 7 August 2024) using the term “High altitude” and downloaded the GSE52209 and GSE133702 chip datasets. The original dataset was processed through quality control, alignment, expression quantification and differential expression analysis to obtain a differential dataset, which was then visualized using the ggplot2 package in R for volcano plot creation. DEGs with *p* < 0.05 and |log2FC| > 0.5 were considered significant. The GSE103927 dataset was used to immune infiltration analysis.

### 4.2. PPI Network Construction

The PPI network of common targets was constructed using the String database (http://string-db.org, accessed on 10 August 2024). Interactions with a combined score of over 0.4 were considered statistically significant. The Cytoscape 3.10.2 software was used to visualize the PPI network. Molecular complex detection (MCODE) is a Cytoscape plugin that finds tightly connected protein clusters (node score cutoff = 0.2, degree cutoff = 2, core threshold K = 2, max. depth = 100).

### 4.3. Immune Cell Infiltration-Related Analysis

To estimate the relative abundances of the immune cell types, the CIBERSORT algorithm, which is a gene expression-based deconvolution algorithm that identifies 547 immune cell-related genes, was applied. Using the CIBERSORT package, we calculated the relative proportions of 22 immune cell types in the control and HH 7-day groups, displayed in a bar chart. *p* < 0.05 was considered to be statistically significant.

### 4.4. Functional Enrichment Analysis

Gene Ontology (GO) term and Kyoto Encyclopedia of Genes and Genomes (KEGG) pathway enrichment analyses of DEGs were performed using the Sangerbox (http://www.sangerbox.com/, accessed on 15 August 2024) tool, showing the function and enrichment pathways of the differentially variable gene sets. Statistical significance was determined using an adjusted *p* value of 0.05.

### 4.5. Correlation Analysis between Hub Genes and Immune Cells

The relationship between the hub genes and the immune cells was revealed by Pearson correlation analysis.

### 4.6. Potential Drug Prediction

HERB (http://herb.ac.cn/, accessed on 23 August 2024) is a high-throughput experiment- and reference-guided database of traditional Chinese medicine that can help researchers to screen for highly relevant drugs for diseases. We found target-related ingredients and selected “Database Mining” or “Reference Mining” items.

### 4.7. Molecular Docking

Molecular docking is one of the most important means of virtual screening for its capacity to accurately predict the conformation of small molecule ligands within the appropriate target binding site and to estimate the most favorable orientation and the binding affinity [54]. The 3D molecular structures of JAK2- and TNFSF13B (PDB ID: 2B7A;1XU2)-related active ingredients (jasminoidin CID: 107848; isoliquiritigenin CID: 638278; dicumarol CID: 54676038; artemotil CID: 3000469) were obtained in PubChem (https://www.ncbi.nlm.nih.gov/, (accessed on 1 September 2024)), and the crystal structures of the targets were acquired from the PDB Database (https://www.rcsb.org/, (accessed on 1 September 2024)). The active ingredients were converted to .pdbqt format, and the target protein was imported into Pymol, preprocessed to add hydrogen atoms and assign charges. Then, molecular docking was performed using Autodock Vina 1.2.5. This software was used to convert the “.pdb” format of the natural compounds and target files to the “.pdbqt” format and construct the protein structure docking box grid (30 × 30 × 30 xyz points) around the binding site. The docking parameters are indicated in Table 1 and display the highest-scoring docking results with Pymol.24.

## 5. Conclusions

This study integrated systems bioinformatics methods to screen for changes in immune cell subtypes and their associated targets in AMS. It also sought potential therapeutically effective candidate natural compound monomers. This provides a reference for the basic research and clinical application of acute mountain sickness.

## Figures and Tables

**Table 1:** Molecular docking results.

Compound	Key Targets	Docking Score (kcal mol^−1^)
Jasminoidin	JAK2	−7.3
Isoliquiritigenin	JAK2	−8.2
Dicumarol	TNFSF13B	−7.5
Artemotil	TNFSF13B	−7.6

**Table 2:** The lengths of the hydrogen bonds.

Target	Compound	Residue	AA	Distance
JAK2	Jasminoidin	858	GLY	2.84
		860	PHE	3.62
		861	GLY	2.03
		936	SER	2.31
		936	SER	2.57
		981	ASN	2.27
		981	ASN	2
JAK2	Isoliquiritigenin	857	LYS	4.04
		882	LYS	4.03
		932	LEU	2.96
TNFSF13B	Dicumarol	182	GLU	3.18

## Data Availability

The data generated in the present study may be requested from the corresponding author on reasonable request.

## Supplementary Materials

The following supporting information can be downloaded at: https://www.mdpi.com/article/10.3390/ijms252011311/s1.
