# Supplementary material for: Exploring the Blood Biomarkers and Potential Therapeutic Agents for Human Acute Mountain Sickness Based on Transcriptomic Analysis, Inflammatory Infiltrates and Molecular Docking
Source: Int J Mol Sci. 2024 Oct 21;25(20):11311. doi: 10.3390/ijms252011311 (PMC11508554; doi:10.3390/ijms252011311)
Supplement: Supplementary file 1 [file ijms-25-11311-s001.zip › ijms-3250073-supplementary.pdf]

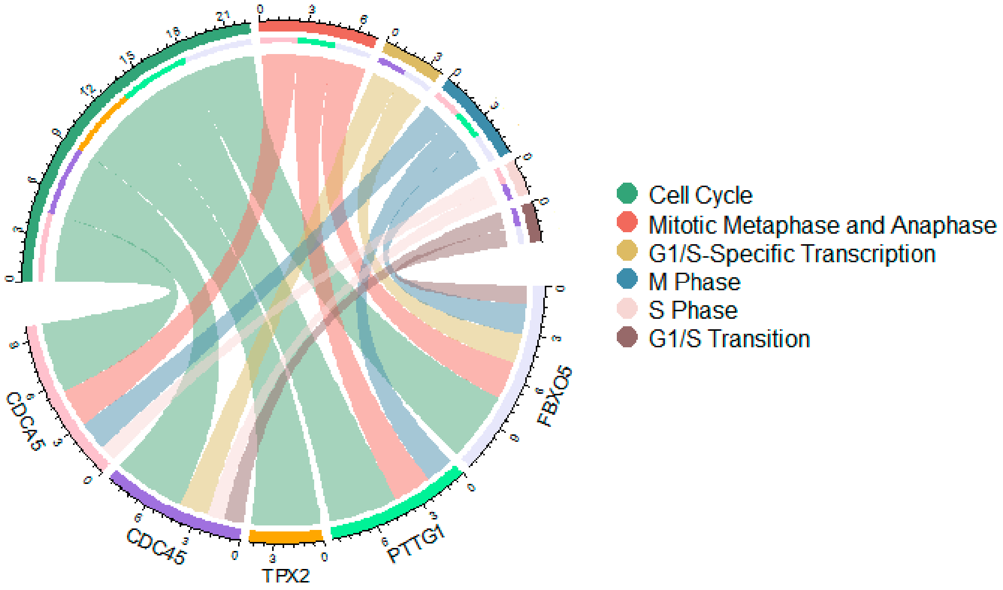

Figure S1.Functional pathway enrichment analysis of the Cluster 1 genes.

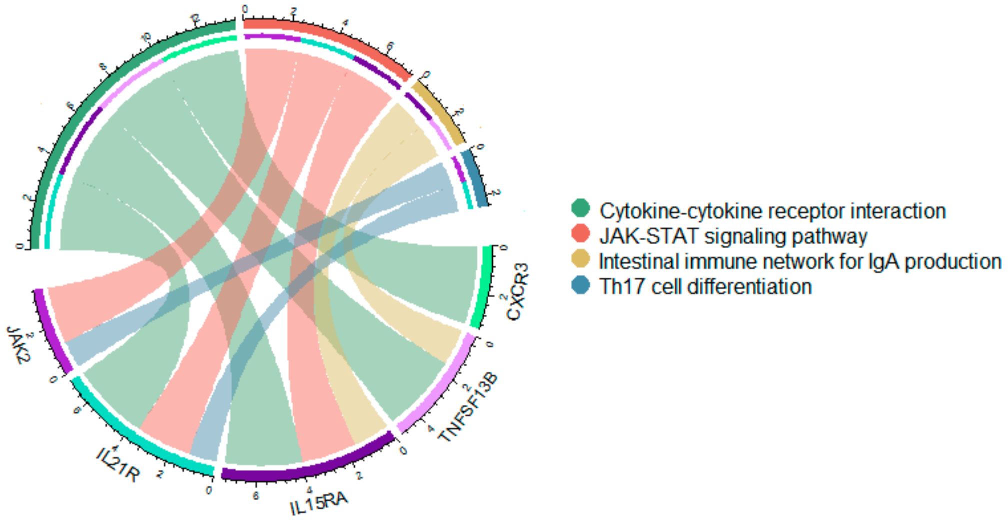

Figure S2.Functional pathway enrichment analysis of the Cluster 2 genes.
